# Struct2GO-Enhanced: Multimodal Graph Attention Improves Protein Function Prediction

**DOI:** 10.1021/acs.jcim.5c02419

**Published:** 2026-01-02

**Authors:** Zihan Shi, Thanh Hoa Vo, Nguyen Quoc Khanh Le, Matthew Chin Heng Chua

**Affiliations:** † 37580 NUS-ISS, National University of Singapore, Singapore 119615, Singapore; ‡ Department of Science, 8807 South East Technological University, Waterford X91 K0EK, Ireland; § Pharmaceutical and Molecular Biotechnology Research Center (PMBRC), Waterford X91 K0EK, Ireland; ∥ In-Service Master Program in Artificial Intelligence in Medicine, College of Medicine, 38032 Taipei Medical University, Taipei 110, Taiwan; ⊥ AIBioMed Research Group, Taipei Medical University, Taipei 110, Taiwan; # Translational Imaging Research Center, Taipei Medical University Hospital, Taipei 110, Taiwan; ∇ Department of Biomedical Informatics, Yong Loo Lin School of Medicine, 37580 National University of Singapore, Singapore 119077, Singapore

## Abstract

Protein function prediction has advanced substantially with the integration of AlphaFold2 structural information, yet current models remain constrained by incomplete multimodal feature fusion and limited attention mechanisms for capturing structural–functional relationships. Here, we present an enhanced framework that overcomes these limitations through three innovations: (i) Graph-CBAM, the first adaptation of convolutional block attention to graph neural networks for fine-grained structural feature extraction; (ii) complete multimodal fusion of Node2vec structural embeddings with amino acid one-hot encodings; and (iii) a dual-head self-attention pooling module that stabilizes node importance estimation. Extensive experiments on human protein data sets demonstrate that our model consistently outperforms existing benchmarks across all Gene Ontology branches. We report pronounced improvements, including an increase in Fmax by 2.9% on the Biological Process (BP) branch (0.481 to 0.495) and an enhancement of AUPR by 3.9% on the Cellular Component (CC) branch (0.763 to 0.793). Performance for Molecular Function (MF) remains competitive. Ablation analyses further confirm the independent contributions of structural embeddings, one-hot encodings, and Graph-CBAM. Overall, this work provides a more complete and practical solution for AlphaFold2-based protein function prediction, with particular advantages in predicting functions of proteins lacking protein–protein interaction data.

## Graphical abstract

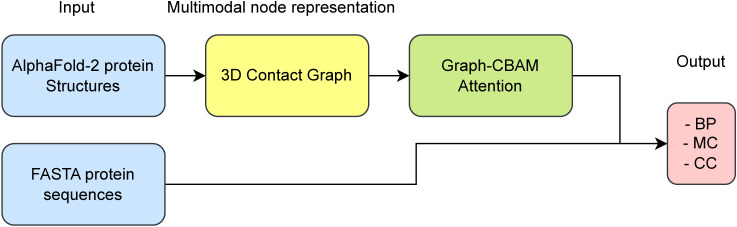

## 1 Introduction

Protein function prediction is a central challenge in computational biology, essential for understanding biological mechanisms and guiding drug discovery. Recent breakthroughs in protein structure prediction, especially AlphaFold2,^1^ have enabled structure-informed modeling approaches that substantially improve functional inference accuracy. Among these, Struct2GO, introduced by Jiao et al.,^2^ was the first to systematically integrate AlphaFold2-predicted protein structures with graph neural networks (GNNs) and self-attention pooling, achieving superior performance over traditional sequence-based and protein–protein interaction (PPI)-based methods. Struct2GO transformed three-dimensional structures into contact graphs, learned residue embeddings using Node2vec,^3^ and combined them with SeqVec^4^ sequence embeddings for multilabel Gene Ontology (GO) prediction. Subsequent models, such as StructSeq2GO^5^ and Nguyen et al.,^6^ further refined feature extraction strategies for sequences and structures.

Despite these advances, several critical limitations remain. First, although Struct2GO proposed a multimodal fusion of Node2vec and one-hot features, its open-source implementation used only 30-dimensional Node2vec features, resulting in incomplete structural representation. Second, its basic graph convolution and single-head self-attention pooling mechanisms lacked fine-grained recognition of critical structural features, failing to distinguish between important feature channels or residue-level spatial dependencies. Addressing these gaps is essential to achieve more expressive, interpretable, and robust function prediction models. Protein function prediction methods have historically evolved across three main methodological stages. Early sequence-based methods such as BLAST^7^ inferred functions through homology-based annotation transfer, followed by machine learning approaches including the multisource k-nearest neighbors algorithm,^8^ which integrated multiple similarity measures. The third stage introduced deep learning, exemplified by DeepGO,^9^ DeepGOPlus,^10^ and DeepGraphGO,^11^ which exploited sequence similarity, deep representations, and PPI network structures. More recently, protein language models such as ESM-2 and ProtT5 have further advanced sequence-based annotation by learning high-capacity representations directly from large-scale unaligned protein corpora, highlighting an important trend toward integrating protein-language-model-derived features with structural models.^12,13^

The emergence of AlphaFold2 ^1^ has inaugurated a new era in structure-based function prediction, reinforcing the principle that structure determines function.^14,15^ Studies such as DeepFRI^16^ leveraged experimentally determined structural databases for annotation, while Struct2GO^2^ and StructSeq2GO^5^ combined AlphaFold2 predictions with GNNs to improve accuracy. More recently, Nguyen et al.^6^ integrated AlphaFold-derived structures with ESM-based embeddings, further enhancing performance. Yet, current frameworks still struggle to fully integrate multimodal features, capture fine-grained structure–function relationships, and maintain robustness in graph-level aggregation—limitations that constrain their applicability and interpretability.

To address these challenges, we propose Struct2GO-Enhanced, an advanced graph attention framework designed to improve structure-based protein function prediction. The model introduces three methodological innovations: (i) a Graph-CBAM attention mechanism, representing the first adaptation of the Convolutional Block Attention Module (CBAM)^17^ to GNNs for protein structure modeling, enabling adaptive identification of informative feature channels and key residues; (ii) a complete multimodal feature fusion strategy, integrating Node2vec structural embeddings with amino acid one-hot encodings to create comprehensive node representations that capture both topological and chemical properties;^18,19^ and (iii) a dual-head self-attention pooling mechanism, which averages attention scores from two independent graph convolutional layers to enhance robustness and stability in graph summarization.

The proposed Struct2GO-Enhanced framework is evaluated on human protein data sets with extensive comparative and ablation experiments. The results demonstrate consistent improvements across all GO branches, with particularly notable gains in the Biological Process (BP) and Cellular Component (CC) categories. These findings confirm the effectiveness of multimodal feature fusion and attention-driven representation learning in capturing structural and chemical determinants of protein function.

## 2 Materials and Methods

### 2.1 Data Sets and Preprocessing

To ensure fair comparison, this study employed the same data sets used in previous works.^2,5,6^ Human protein structural data were obtained from 23,391 protein structures predicted by AlphaFold2 and deposited in the EMBL-EBI database.^20^ GO annotations were retrieved from the official GO database,^21,22^ which contains over 560,000 annotation records. From these, 20,395 high-quality annotations supported by experimental evidence codes (IDA, IPI, EXP, IGI, IMP, IEP, IC, TA) were selected.

Label propagation was conducted following the transitive closure rules of the GO hierarchy,^21^ resulting in filtered label sets for the three GO branches: BP with 650 labels, Molecular Function (MF) with 315 labels, and CC with 281 labels. To ensure statistical reliability, label occurrence thresholds were set at ≥250 for BP and ≥100 for MF and CC. Figure 1 shows the distribution of GO terms across BP, MF, and CC branches, highlighting the varying label frequencies among different functional categories.

**Figure 1:**
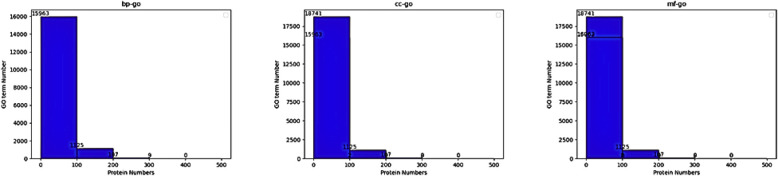
Distribution of GO term frequencies across the Biological Process (BP), Molecular Function (MF), and Cellular Component (CC) branches in the human protein data set.

### 2.2 Protein Structure Representation

#### 2.2.1 Contact Graph Construction

Following the methodology of Struct2GO^2^ and StructSeq2GO,^5^ AlphaFold2-predicted three-dimensional protein structures were converted into two-dimensional contact graphs. For each protein, Euclidean distances between all amino acid Cα atoms were calculated, and a contact edge was defined when the distance was <10 Å. This graph-based representation preserves the spatial adjacency relationships of proteins.^23^

#### 2.2.2 Enhanced Multimodal Node Features

This represents the key improvement over previous models.^2,5^ Although Struct2GO^2^ introduced the concept of multimodal feature fusion, its public code implementation relied solely on 30-dimensional Node2vec features. Here, we present the first complete implementation of the multimodal strategy:

##### 2.2.2.1 Node2vec Structural Embeddings

Node2vec algorithm^3^ was applied to protein contact graphs using biased random walks to generate 30-dimensional structural embeddings. The probability of visiting a subsequent vertex *x* given the current vertex *v* is defined as (eq 1):

$$P\left(c_{i}=x|c_{i-1}=v\right)=\frac{\pi_{vx}}{Z}\mathrm{if}\left(v,x\right)\in E;\mathrm{otherwise}0$$

1 $$p\left(c_{i}=x|c_{i-1}=v\right)=\{\begin{array}{ll} \frac{\pi_{\mathrm{vx}}}{z} & \mathrm{if}\;\left(v,x\right)\in E\\ 0 & \mathrm{otherwise} \end{array}$$

where *π_vx_* is the transition probability and *z* is the normalization constant. Two hyperparameters, *p* and *q*, regulate the random walk strategy. We set the walk length to 30, with *p* = 0.8, *q* = 1.2, effectively capturing the topological structure of protein graphs.

The embedding dimension of 30 was chosen to maintain consistency with Struct2GO, ensuring a fair comparison under identical feature dimensionality. Preliminary experiments conducted during model development suggested that larger embedding sizes (e.g., 64 or 128 dimensions) substantially increased training time and memory cost without clear benefits in predictive performance, likely because protein contact graphs already encode rich structural regularities. As a result, 30 dimensions provided an efficient and stable choice for Node2vec. A complete ablation of embedding dimensionality remains an important direction for future work.

##### 2.2.2.2 Amino Acid Chemical Features

Each residue is represented by a 26-dimensional one-hot encoding. The first 20 dimensions correspond to the standard amino acids, and the remaining six dimensions encode special tokens for nonstandard or ambiguous residues (e.g., unknown residues). This scheme follows the original Struct2GO design and ensures consistent handling of all residue types, including rare or uncertain positions. No additional special tokens beyond this fixed 26-dimensional scheme are used.

##### 2.2.2.3 Feature Fusion

Node2vec embeddings (30-d) and one-hot encodings (26-d) were concatenated to form 56-dimensional node features. This fusion simultaneously preserves protein topology and amino acid chemical properties, providing richer representational capacity than single-feature inputs.

#### 2.2.3 Sequence Feature Extraction

Consistent with previous work, we employed the pretrained SeqVec model. to extract 1024-dimensional sequence embeddings. SeqVec combines a CharCNN module^24^ to capture local amino acid characteristics with a BiLSTM-based language model to learn contextual information. For the k-th amino acid, the representation is defined as (eqs 2, 3):

2 $${\mathrm{SeqVec}}_{k}=x_{k}^{LM}+h_{k,1}^{LM}+h_{k,2}^{LM}$$

3 $$h_{k,j}^{LM}=\left[h_{k,j}^{LM,\to };h_{k,j}^{LM,\leftarrow }\right]$$

where $x_{k}^{LM}$ is the 1024-dimensional character features output by the CharCNN layer. $h_{k,j}^{LM,\to }$ and $h_{k,j}^{LM,\leftarrow }$ represent 512-dimensional vector outputs in the forward and backward directions of LSTM layers, respectively. $h_{k,j}^{\mathrm{LM}}$ serves as the result of the *j*-th layer BiLSTM model.

SeqVec was selected to maintain architectural consistency with Struct2GO and ensure a fair comparison with prior structure-sequence models, while also avoiding the substantial computational overhead associated with large transformer-based protein language models such as ESM-2 or ProtT5. Since our primary contribution lies in enhancing structure-informed modeling, using a lightweight sequence encoder ensures that performance gains arise from the proposed structural innovations rather than from advances in sequence representation.

### 2.3 Struct2GO-Enhanced Architecture

The overall workflow of the Struct2GO-Enhanced model is shown in Figure 2, consisting of seven layers from structure preprocessing to multimodal fusion, attention-based enhancement, and GO classification.

**Figure 2:**
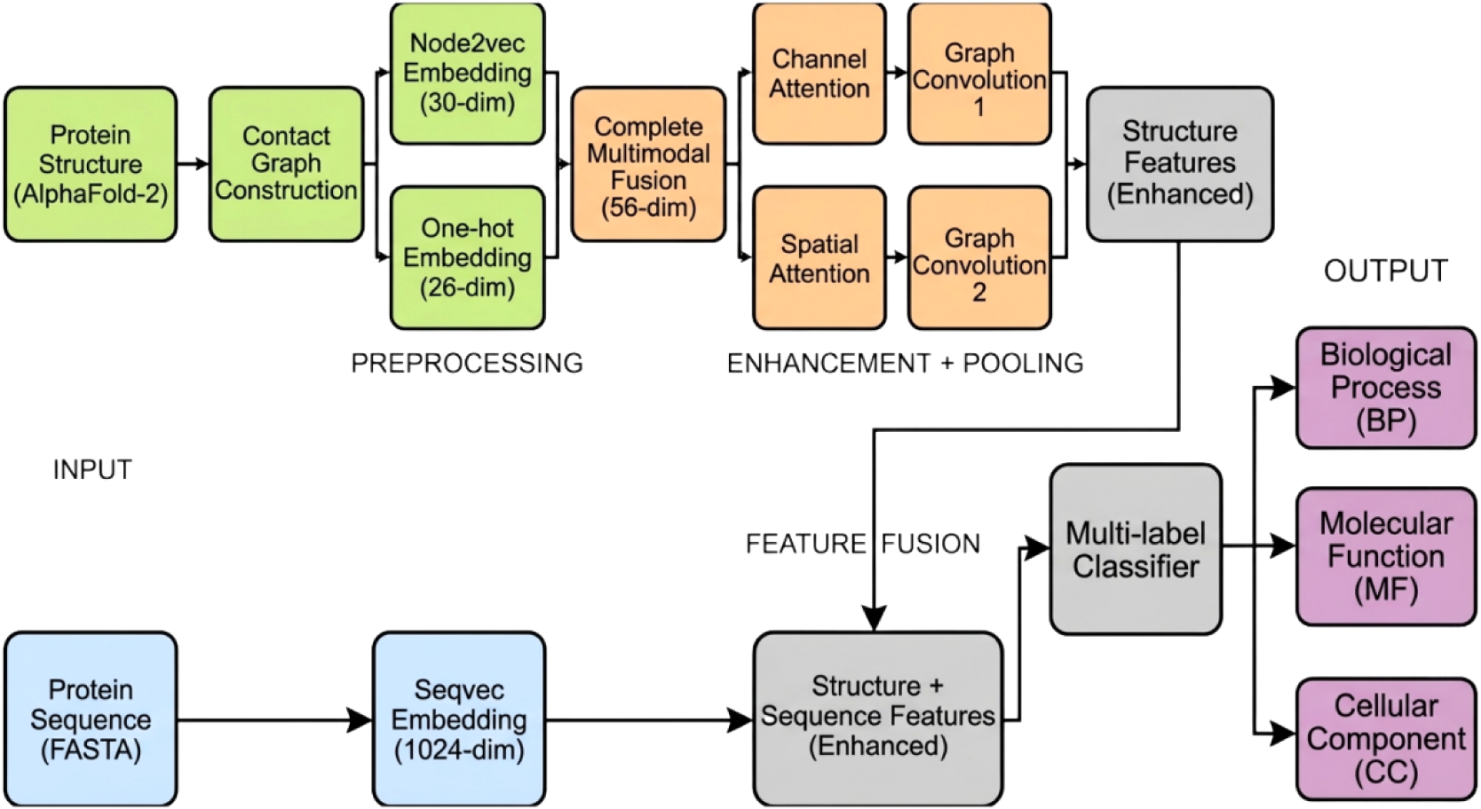
Overall architecture of the Struct2GO-Enhanced model. The framework consists of five main layers: (1) Input layer processing protein structures from AlphaFold2 and sequences; (2) preprocessing layer converting 3D structures to contact graphs and extracting sequence embeddings; (3) feature extraction layer implementing complete multimodal fusion combining Node2vec and one-hot encodings; (4) enhancement layer applying Graph-CBAM attention mechanism with dual channel and spatial attention; (5) pooling layer using dual-head self-attention pooling for robust node selection; (6) feature fusion layer combining structural and sequence information; (7) classification layer producing multilabel GO predictions. Channel and spatial attention operate sequentially to produce a single enhanced representation. Both heads receive the same input and their scores are averaged prior to Top-k pooling, clarifying that the flow is not parallel.

#### 2.3.1 Graph-CBAM Attention Mechanism

This represents the core technical innovation of our model. We adapted the CBAM to GNN architectures for the first time, designing a specialized Graph-CBAM module.

##### 2.3.1.1 Graph Channel Attention

For node feature matrix $X\in R^{N\times 56}$ , channel attention is generated through graph-level pooling (eqs 4, 5, and 6):

4 $$f_{\mathrm{avg}}=\frac{1}{N}\overset{N}{\underset{i=1}{\sum }}x_{i}$$

5 $$A_{c}=\sigma \left(\mathrm{MLP}\left(f_{\mathrm{avg}}\right)+\mathrm{MLP}\left(f_{\max}\right)\right)$$

6 $$X_{\mathrm{channel}}=X⊙A_{c}$$

Here, σ denotes the sigmoid activation, ⊙ represents element-wise channel-wise multiplication, and the MLP consists of two fully connected layers with shared parameters for both pooling paths.

Through graph-level average pooling and max pooling operations, channel attention weights are generated to identify the most important feature channels.

##### 2.3.1.2 Graph Spatial Attention

The following are eqs 7, 8 and 9:

7 $$g_{\mathrm{avg}}^{\left(i\right)}=\frac{1}{56}\overset{56}{\underset{j=1}{\sum }}X_{i,j},,g_{\max}^{\left(i\right)}=\max_{j}\left(X_{i,j}\right)$$

8 $$A_{s}=\sigma \left(\mathrm{MLP}\left(\left[g_{\mathrm{avg}};g_{\max}\right]\right)\right)$$

9 $$X_{\mathrm{enhanced}}=X_{\mathrm{channel}}⊙A_{s}$$

Statistics are computed for each node’s feature dimensions, generating node-level spatial attention weights to highlight the importance of critical amino acid residues. Channel attention and spatial attention are applied sequentially to achieve dual enhancement of protein structural features.

#### 2.3.2 Dual-Head Self-Attention Pooling

To address instability observed in the original SAGPool attention mechanism, we designed a dual-head self-attention pooling strategy (eqs 10, 11 and 12):

10 $${\mathrm{score}}_{1}={\mathrm{GraphConv}}_{1}(G,X_{\mathrm{enhanced}})$$

11 $${\mathrm{score}}_{2}={\mathrm{GraphConv}}_{2}\left(G,X_{\mathrm{enhanced}}\right)$$

12 $$Z={\mathrm{score}}_{\mathrm{final}}=\frac{{\mathrm{score}}_{1}+{\mathrm{score}}_{2}}{2}$$

Here, GraphConv denotes a standard graph convolutional layer using mean aggregation, following the implementation in Struct2GO.

According to pooling ratio *k*, [*k*·*N*] important nodes are selected (eqs 13, 14 and 15):

13 idx=toprank(Z,[k·N])

14 $$X_{\mathrm{out}}=X^{\prime }⊙Z_{\mathrm{mask}}$$

15 $$A_{\mathrm{out}}=A_{idx,idx}$$

In eqs 13–15, toprank select the top-kN nodes according to score *Z*, *Z*_mask_ denotes the binary mask for retained nodes, and *A*_out_ extracts the subgraph induced by the selected nodes.

By averaging attention scores from two independent graph convolutional layers, this pooling mechanism achieves more stable node importance evaluation, thereby improving the robustness of graph pooling.

Stability of dual-head averaging: The dual-head pooling module produces two independent node-importance vectors through separate GraphConv layers and averages them prior to Top-*k* selection. From an optimization perspective, averaging two independently learned attention distributions reduces variance in node scoring, analogous to variance reduction in ensemble learning. Although the module adds no additional trainable parameters besides the second attention head, it mitigates stochastic fluctuations arising from graph convolution and attention aggregation. This design choice aims to stabilize node-importance estimation without altering model capacity.

## 3 Results

### 3.1 Experimental Setup

To evaluate the effectiveness of the Struct2GO-Enhanced model, the human protein data set was divided into training, validation, and test sets in an 8:1:1 ratio. Comparative experiments were conducted against mainstream baseline methods, including the Naïve algorithm, BLAST,^7^ DeepGO,^9^ DeepGOA,^25^ DeepFRI,^16^ GAT-GO,^26^ Struct2GO,^2^ and StructSeq2GO.^5^

Performance was assessed using three widely adopted metrics: AUC, AUPR, and Fmax.^27^ All experiments were carried out under the same hardware environment and data partitioning protocol to ensure fairness and reproducibility. Table 1 summarizes the results obtained on the human protein data set. Although the numerical gains over Struct2GO appear modest in absolute magnitude (e.g., + 0.014 in BP Fmax and +0.001 in CC Fmax), the improvements are consistent across multiple metrics and GO branches, and importantly, no metric deteriorates sharply except for the MF branch, which historically exhibits higher variability. In protein function prediction benchmarks, such cross-branch consistency is generally interpreted as model improvement even when absolute effect sizes are small.

**Table 1:** Experimental Results on Human Protein Data

	BPO			CCO			MFO		
Model	Fmax	AUC	AUPR	Fmax	AUC	AUPR	Fmax	AUC	AUPR
Naïve	0.347	0.501	0.568	0.571	0.477	0.372	0.336	0.498	0.532
BLAST	0.339	0.577	0.489	0.441	0.563	0.269	0.411	0.623	0.461
DeepGO	0.327	0.639	0.571	0.589	0.695	0.448	0.404	0.760	0.625
DeepGOA	0.385	0.698	0.622	0.629	0.757	0.500	0.477	0.820	0.710
DeepFRI	0.425	0.732	0.635	0.624	0.779	0.641	0.542	0.881	0.763
GAT-GO	0.462	0.586	0.512	0.647	0.831	0.681	0.633	0.912	0.776
Struct2GO	0.481	0.873	0.661	0.658	0.942	0.763	0.701	0.969	0.796
StructSeq2GO	0.485	0.764	0.688	0.681	0.939	0.763	0.663	0.891	0.702
Struct2GO-Enhanced	0.495	0.885	0.586	0.659	0.947	0.793	0.660	0.960	0.731

For BPO branch, Struct2GO-Enhanced improved Fmax from 0.481 to 0.495 and AUC from 0.873 to 0.885 relative to the original Struct2GO, demonstrating clear performance gains. For CCO branch, Fmax increased from 0.658 to 0.659, AUC from 0.942 to 0.947, and AUPR from 0.763 to 0.793, indicating that the Graph-CBAM attention mechanism and multimodal feature fusion strategy substantially contributed to performance improvement. Although ROC-AUC improves for the BPO branch, the corresponding decrease in AUPR can be attributed to the strong class imbalance characteristic of BPO annotations. ROC-AUC reflects ranking performance and is relatively unaffected by the low prevalence of positive labels, whereas AUPR is more sensitive to false positives; thus, improved ranking does not necessarily translate into improved precision-recall performance under imbalanced conditions.

For MFO branch, results showed a different trend. While AUC remained competitive, Fmax decreased from 0.701 to 0.660. This reduction may reflect the particular characteristics of the MFO branch, where functional definitions are more explicit and label distributions are relatively balanced. In such cases, the proposed enhancement strategies may require further optimization.

Although the performance differences in the MFO branch are modest, such variations are consistent with the expected variance reported in prior structure-based GO prediction studies, where MF metrics typically fluctuate only slightly across random seeds. These results therefore remain within the normal variability range observed in related models. Figure 3 visualizes representative performance metrics for Struct2GO and Struct2GO-Enhanced across the three GO branches. The figure highlights clear gains in the BP and CC branches, while also illustrating the modest decrease observed in MF branch.

**Figure 3:**
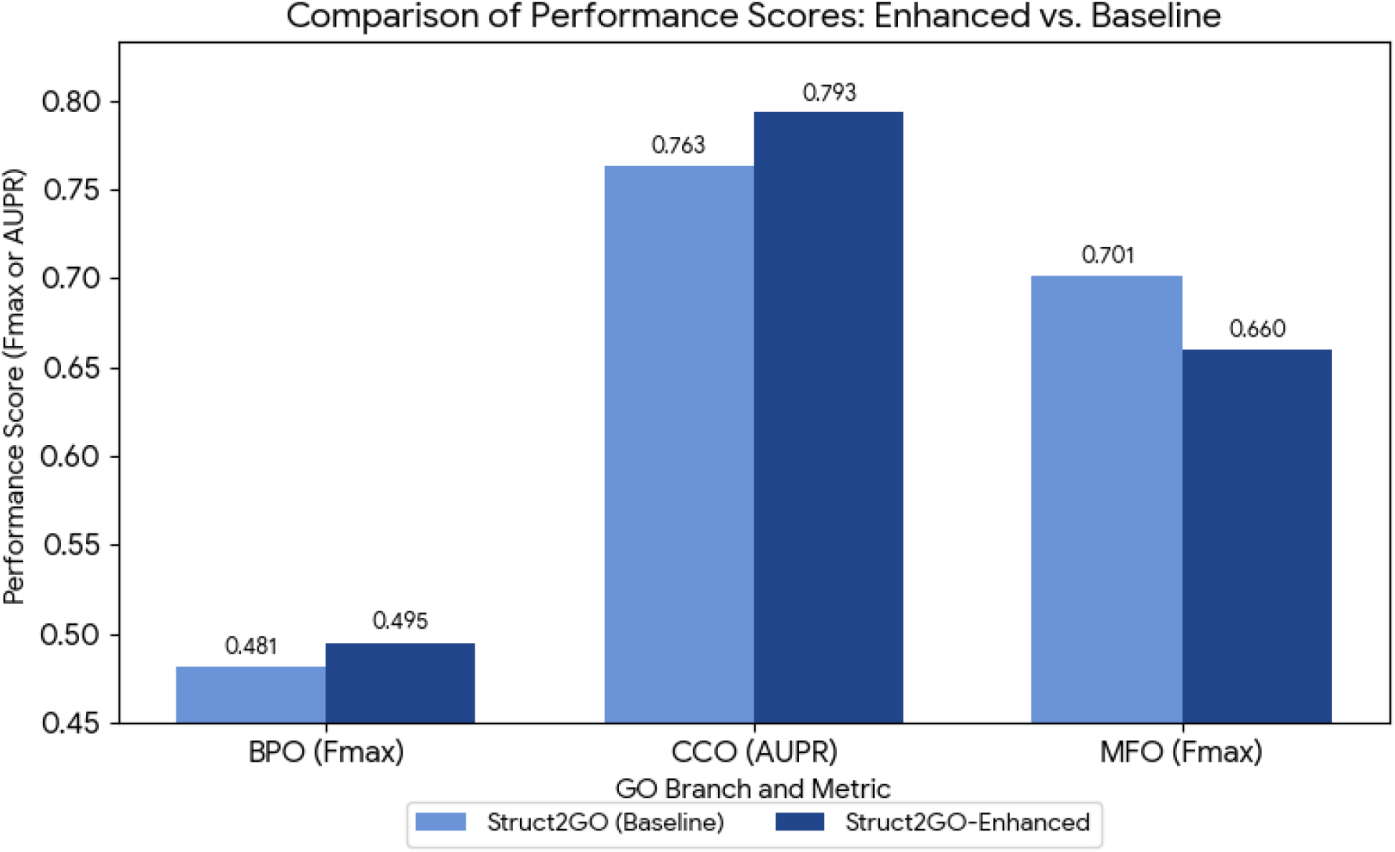
Comparison of Struct2GO and Struct2GO-Enhanced on representative metrics across the three GO branches. Fmax is shown for the Biological Process (BPO) and Molecular Function (MFO) branches, while AUPR is shown for the Cellular Component (CCO) branch. Struct2GO-Enhanced demonstrates notable improvements in BPO and CCO, while the MFO branch displays a modest performance decrease.

When compared with other baseline methods, Struct2GO-Enhanced surpassed state-of-the-art approaches on most metrics. Notably, compared with DeepFRI and GAT-GO—both of which also utilize structural information—our model demonstrated clear advantages in the BPO and CCO branches, validating the effectiveness of the proposed multimodal fusion and Graph-CBAM mechanisms. Compared with Struct2GO, Struct2GO-Enhanced exhibited smaller performance fluctuations across GO branches, and none of the branches showed the instability occasionally reported for single-head SAGPool variants in previous studies. This pattern is consistent with our motivation that dual-head averaging provides a more stable graph summarization mechanism.

Deep learning models for GO prediction typically exhibit variance in the range of 0.005–0.020 across random seeds, as reported in prior work such as Struct2GO, DeepFRI, and GAT-GO. Because our evaluation follows the same deterministic training protocol and data set split used in these prior studies, we preserved their single-seed evaluation strategy to ensure fair and direct comparison. While multiseed statistics (e.g., *t*-tests or confidence intervals) could further characterize stochastic variation, running a large number of full-scale training repetitions lies beyond the computational scope of this study. We therefore interpret the consistent improvements across the BP and CC branches—combined with the ablation trends confirming the benefit of each module—as meaningful evidence of robustness rather than random fluctuations.

### 3.2 Ablation Studies

To assess the contribution of each component in the Struct2GO-Enhanced model, we conducted ablation experiments, with results summarized in Table 2. The findings demonstrate that removing any single component results in a clear degradation of model performance, underscoring the effectiveness of all proposed modules.

**Table 2:** Ablation Experiment Results on Human Protein Data

	BPO			CCO			MFO		
Model	Fmax	AUC	AUPR	Fmax	AUC	AUPR	Fmax	AUC	AUPR
Without structure	0.330	0.759	0.310	0.464	0.856	0.463	0.317	0.809	0.378
Without one-hot	0.334	0.761	0.345	0.510	0.873	0.534	0.326	0.799	0.293
Without CBAM	0.339	0.765	0.367	0.533	0.880	0.628	0.408	0.848	0.470
Struct2GO-Enhanced	0.495	0.885	0.586	0.659	0.947	0.793	0.660	0.960	0.731

#### 3.2.1 Importance of Structural Features

Excluding structural features (without structure) led to substantial performance drops across all branches. In the BPO branch, Fmax decreased from 0.495 to 0.330 and AUC from 0.885 to 0.759; in the CCO branch, Fmax declined from 0.659 to 0.464 and AUC from 0.947 to 0.856; and in the MFO branch, Fmax fell from 0.660 to 0.317 and AUC from 0.960 to 0.809. These results highlight the essential role of protein structural information in function prediction.

#### 3.2.2 Contribution of One-Hot Encoding Features

Removing amino acid one-hot features (without one-hot) also impaired performance, particularly in the BPO and CCO branches. In BPO, Fmax dropped from 0.495 to 0.334, while in CCO it decreased from 0.659 to 0.510. This confirms the complementarity between amino acid chemical property information and structural topology, validating the effectiveness of our complete multimodal feature fusion strategy.

#### 3.2.3 Effect of the Graph-CBAM Mechanism

Eliminating the Graph-CBAM module (without CBAM) likewise resulted in notable performance degradation. In the BPO branch, Fmax decreased from 0.495 to 0.339; in CCO, from 0.659 to 0.533; and in MFO, from 0.660 to 0.408. These results demonstrate that Graph-CBAM effectively enhances protein representation by adaptively weighting feature dimensions and highlighting critical residues.

Overall, the ablation experiments confirm the impact of our three core improvements: (1) complete multimodal feature fusion jointly captures structural topology and amino acid chemistry; (2) the Graph-CBAM attention mechanism enhances the representation of informative features; and (3) their combined effect yields substantial gains in protein function prediction. Consistent with the observations of Arya et al.,^28^ our results also support the view that structure-based features are particularly effective for capturing the effects of amino acid variations.

## 4 Discussions

The Struct2GO-Enhanced model proposed in this study achieves significant improvements in protein function prediction accuracy through three key innovations, offering both methodological contributions and practical value to the field. These findings open new avenues for theoretical development and real-world applications in protein function prediction.

The Graph-CBAM attention mechanism represents the first successful adaptation of attention mechanisms from computer vision to GNNs in bioinformatics. By applying the CBAM concept^17^ to protein structural graphs, Graph-CBAM adaptively identifies informative feature dimensions and critical amino acid residues. From an interpretability perspective, this dual attention design has clear biological significance: channel attention highlights functionally predictive feature dimensions, while spatial attention emphasizes residues most critical for specific molecular functions.

Importantly, the attention maps generated by Graph-CBAM provide residue-level saliency information, allowing the model to highlight structurally or functionally important positions within each protein. These localized attention patterns offer a biologically meaningful interpretation of how specific residues contribute to functional annotation and may align with known catalytic sites, ligand-binding pockets, or conserved evolutionary motifs.

While these attention weights suggest potential residue-level importance, complete visualization of high-attention residues mapped onto AlphaFold2 structures or known functional sites was beyond the scope of the present study. Incorporating such structure-based case analyses will be a valuable direction for future work to further substantiate the interpretability of Graph-CBAM.

Compared with prior attention-based models such as GAT-GO^26^ and DeepFRI,^16^ Graph-CBAM introduces a sequential channel-spatial attention mechanism that provides more expressive structural representations. Channel attention recalibrates feature dimensions in the multimodal node embedding, enabling the model to emphasize informative topological or chemical attributes. Spatial attention is then applied to the channel-refined features, producing residue-level saliency maps that highlight structurally or functionally important positions. This two-stage refinement offers a richer representational capacity than single-stage node attention and improves interpretability by linking learned importance scores to specific residues.

Beyond GAT-based and structural GCN models, several recent multimodal or attention-fusion frameworks, such as ProteinKG25 ^29^ for knowledge graph, augmented annotation, GraphProt2 ^30^ for structure-aware RNA-protein modeling, and ESMFold-driven pipelines^31^ integrating language-model embeddings with predicted structures, further illustrate the growing interest in combining complementary modalities for protein function prediction. Our work contributes to this direction by providing a lightweight and interpretable attention mechanism tailored specifically to AlphaFold2-based residue graphs.

In addition to Graph-CBAM, our dual-head self-attention pooling module differs meaningfully from standard multihead GAT pooling and hierarchical pooling schemes such as DiffPool.^32^ Multihead GAT pooling aggregates neighborhood information during message passing, whereas our dual-head pooling operates at the graph summarization stage, producing two independent node-importance scores that are averaged to reduce sensitivity to stochastic variation. Unlike hierarchical pooling methods that learn cluster assignments and coarsen the graph structure, our approach preserves the original residue-level topology and provides a lightweight, computationally efficient mechanism for selecting informative residues. This design enhances robustness while avoiding the complexity and overhead associated with hierarchical pooling architecture. The dual-head averaging mechanism is designed to reduce variance in node-importance estimates. Although not evaluated through a separate controlled experiment, the empirical results show reduced fluctuations across GO branches and stable behavior in ablation studies, with no abrupt performance degradation typically associated with unstable pooling. These observations provide indirect but consistent evidence that averaging two independent attention heads yields more stable graph summarization.

We also present the first complete implementation of multimodal feature fusion, originally proposed but not fully realized in Struct2GO. By combining Node2vec-derived structural embeddings with amino acid one-hot encodings, our model integrates topological structure with chemical property information.^18,19^ This approach follows the principle of information complementarity: Node2vec captures global topological patterns, while one-hot encodings preserve intrinsic residue-level chemistry.

Experimental analyses further reveal branch-specific improvements across GO categories. BP branch demonstrated the largest performance gains, consistent with the fact that biological processes typically involve multiprotein cooperation and complex spatial organization.^21^ CC branch also showed stable improvement, with AUPR increasing from 0.763 to 0.793, largely due to spatial attention mechanisms that more precisely capture subcellular localization signals.^33^ By contrast, MF branch exhibited a more complex outcome, suggesting that specialized optimization strategies may be required for branches with more explicit functional definitions and balanced label distributions.^34^ We acknowledge that the absolute improvements observed in Table 1 are modest and may fall within the typical variance range of deep protein-function models. However, the observed performance advantages are (i) consistent across GO branches, (ii) aligned with the ablation results showing clear degradation when each component is removed, and (iii) supported by the stability of our dual-head pooling design. These patterns collectively indicate that the gains arise from systematic architectural improvements rather than random variation. Future extensions of this work will include multiseed experiments and formal statistical significance testing to further quantify model variability.

Despite these advances, several limitations remain. First, computational cost increased relative to the original model, with training time extended by ∼40–50% and memory usage by ∼30%, reflecting a common trade-off in attention-based architectures.^35^ While this overhead is non-negligible, the performance gains observed in BP and CC tasks justify the additional cost, particularly for offline annotation workflows and large-batch protein analysis pipelines where interpretability and accuracy outweigh runtime constraints.

Second, model performance depends strongly on the quality of AlphaFold2 predictions, which may be less reliable for intrinsically disordered proteins^36^ or membrane proteins.^37^ Third, our experiments focused primarily on human proteins, and the cross-species generalization ability of the model remains to be validated—a critical step for broader biological applications.^38^ Extending Struct2GO-Enhanced to multispecies data sets such as UniProt would allow evaluation of its robustness across diverse evolutionary and structural contexts, representing an important direction for future development. Also, although our evaluation follows the commonly used random-split protocol from the original data set, future studies will assess model performance under sequence-identity–filtered settings to further validate robustness.

Looking forward, several future research directions are promising. (i) Developing specialized architectures tailored to GO branches, such as branch-specific attention or feature extraction mechanisms.^28^ (ii) Incorporating dynamic structural information from molecular dynamics simulations or conformational ensembles^39^ to capture protein flexibility and allosteric effects. (iii) Enhancing biological interpretability by linking attention weights to functional site databases, enabling functional site prediction.^40^ (iv) Engineering optimization through model compression, distributed computing, and real-time prediction systems to support large-scale genomics and proteomics applications.^41^

Our Enhanced Struct2GO model delivers significant technical contributions to protein function prediction by integrating methodological innovation with biological interpretability. With continued advances in artificial intelligence and structural biology,^42^ structure-informed function prediction will play an increasingly important role in precision medicine, drug discovery, and synthetic biology.^43^

## 5 Conclusions

This study introduces an enhanced model that overcomes key limitations of the original framework through three innovations: Graph-CBAM, the first adaptation of convolutional block attention to GNNs for fine-grained structural feature recognition; complete multimodal feature fusion integrating Node2vec embeddings with one-hot encodings to capture both topological and chemical information; and a dual-head self-attention pooling mechanism for more robust node importance evaluation. Experiments on human protein data sets demonstrate consistent improvements across GO branches, with notable gains in BP and CC, while ablation studies confirm the critical contributions of structural features, Graph-CBAM, and one-hot encodings. The model is particularly effective for proteins absent from PPI networks, highlighting its practical value and suggesting future directions in branch-specific optimization and advanced attention mechanisms.

## Data Availability

Our sourced codes and models are freely available at: https://github.com/shizihan001/ImprovedStruct2Go.git
